# A Scalable Large‐Area Fabrication Strategy for Transparent Polymeric Encapsulated Metagratings

**DOI:** 10.1002/nap2.70224

**Published:** 2026-07-25

**Authors:** Francesca Filograno, Olivier Gauthier‐Lafaye, Adrian Laborde, Jean‐Baptiste Doucet, Vincent Raimbault, Nathalie Bardou, Béatrice Dagens, Giovanna Calò, Giovanni Magno

**Affiliations:** ^1^ Department of Electrical and Information Engineering Polytechnic University of Bari Bari Italy; ^2^ Laboratoire d'analyse et d'architecture des Systèmes (LAAS) Université de Toulouse CNRS Toulouse France; ^3^ Centre de Nanosciences et de Nanotechnologies (C2N) Université Paris‐Saclay CNRS Palaiseau France

**Keywords:** augmented reality display, guide mode resonant metagrating, polymeric and transparent grating, selective color reflection, thermal nanoimprint lithography

## Abstract

In this work, we present the design, fabrication, and experimental demonstration of fully polymeric and transparent guided‐mode resonant (GMR) metagratings for visible‐light operation and integration into augmented reality (AR) systems. The proposed metastructure employs an encapsulated grating architecture in which the periodic pattern is embedded at the substrate–waveguide interface, intrinsically protecting the grating from environmental exposure while preserving the optical functionality. The negative pattern is first transferred by thermal nanoimprint lithography (T‐NIL) into a CYTOP substrate and subsequently backfilled with a high‐index maN resist, providing the refractive‐index contrast required for guided‐mode resonance excitation. Structural characterization by SEM and AFM confirms the successful transfer of the designed geometry, high pattern fidelity and structural uniformity, and excellent reproducibility across independently fabricated samples. Spectroscopic ellipsometry reveals three well‐defined resonances positioned close to the target RGB wavelengths, in good agreement with RCWA simulations, whereas preliminary angle‐dependent reflectance measurements further validate the predicted angular evolution of the guided‐mode resonances. The proposed fabrication strategy demonstrates that thermal nanoimprint lithography provides a robust, scalable, and cost‐effective route for fabricating transparent polymeric GMR metagratings, enabling their implementation in large‐area transparent optical combiners for augmented reality.

## Graphical abstract

Transparent encapsulated polymeric guided‐mode resonant metagratings are fabricated by thermal nanoimprint lithography and experimentally validated through structural and optical characterization. The scalable fabrication strategy enables selective RGB reflection and provides a practical route toward large‐area augmented reality and wavelength‐selective photonic devices.

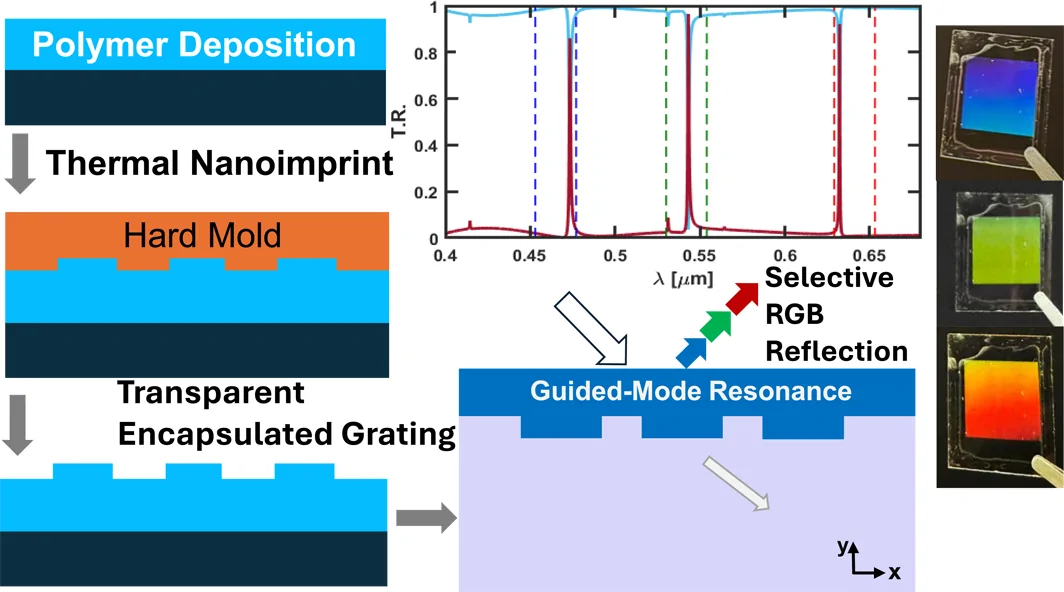

## 1 Introduction

Augmented reality (AR) display systems [1] are increasingly recognized as transformative platforms across consumer electronics, assistive technologies, and biomedical applications. AR displays hold significant potential for clinical and cognitive applications, particularly in supporting aging populations [2] and individuals affected by neurodegenerative diseases or cognitive impairments [3]. In assistive contexts, wearable AR systems can support people's daily lives by improving the execution of everyday activities and facilitating cognitive processes, such as healthcare monitoring and cognitive rehabilitation [4, 5, 6]. The integration of AR lenses into wearable, lightweight, eyeglass‐like platforms remains a major challenge, as it imposes tight constraints on size, power consumption, and especially optical performance. In this framework, a central requirement for AR optics is the ability to selectively reflect narrow bands of the visible spectrum—typically red to convey danger or alert, green for safety, and blue as a neutral baseline or combination point within the red–green–blue (RGB) space—while preserving broadband transmission elsewhere [7, 8]. This functionality enables full‐color virtual image projection onto the retina while maintaining an unobstructed view of the surrounding environment [9].

Several diffractive technologies have been investigated to realize transparent optical combiners for AR systems, including surface‐relief gratings (SRGs), volume holographic gratings (VHGs), polarization volume gratings (PVGs), holographic waveguides [10, 11], resonant waveguide gratings [12], and, more recently, metasurface‐based waveguides capable of high‐performance full‐color AR operation [13]. Each of these approaches not only presents specific advantages but also important limitations. SRGs and resonant waveguide gratings often require demanding nanofabrication processes and, in some cases, high‐index inorganic material platforms [12]; VHGs may suffer from limited angular and spectral bandwidth, whereas PVGs rely on liquid‐crystal‐based architectures and Bragg diffraction mechanisms [10, 11, 14]. These considerations motivate the investigation of alternative AR‐combiner platforms capable of combining optical performance, environmental robustness, and scalable fabrication.

Among the alternative approaches, guided‐mode resonant (GMR) metagratings have emerged as a particularly attractive platform for wavelength‐selective optical devices for their efficiency and versatility [15]. These dielectric metastructures [16] support leaky waveguide modes that can be excited under phase‐matching conditions, producing sharp and tunable reflectance peaks [17, 18]. Compared to multilayer thin‐film filters or plasmonic metasurfaces [19], GMR gratings offer lower absorption losses, high quality factors, and compatibility with flat and transparent substrates, making them ideally suited for near‐eye optics in AR systems [20, 21, 22]. Furthermore, their ability to support multiple resonances within a compact device enables the design of trichromatic reflectors, opening new possibilities for high‐efficiency color routing in planar (and nonplanar) optics [23, 24].

Guided‐mode resonant structures have been extensively investigated for tailoring resonant optical responses through advanced waveguide geometries [25], actively tunable photonic devices based on phase‐transition materials [26], and broadband reflection functionalities [18, 27]. However, the use of GMR devices as transparent optical combiners for augmented reality applications remains comparatively less explored. From a technological perspective, the scalable fabrication of GMR gratings for visible‐light operation remains a major challenge. Indeed, operation in the visible spectral range requires high‐fidelity pattern transfer and precise control of both lateral dimensions and grating depth in order to preserve the designed resonance conditions. Up to now, the critical dimensions required for visible‐range operation have been reliably achieved mainly through electron‐beam lithography (EBL) [28] or nanoimprint lithography (NIL) [29]. Although EBL offers excellent resolution and remains the gold standard for prototyping, it suffers from high cost, long processing times, and it is fundamentally limited in scalability. Thermal nanoimprint lithography (T‐NIL) [30, 31] offers a powerful alternative, enabling direct patterning of sub‐100 nm features over large areas, with low cost and high throughput [32, 33, 34]. Consequently, NIL has been extensively employed for the fabrication of diffraction gratings and nanostructured optical components in a wide range of photonic applications, including distributed‐feedback lasers [35], nanoimprinted grating‐embedded perovskite solar cells [36], plasmonic gratings [37], sawtooth diffraction gratings [38], slanted gratings for AR waveguides [39], roll‐to‐roll large‐area manufacturing approaches for flexible photonic devices [40], full‐color near‐eye‐display waveguides [41], and high‐density diffraction gratings [42].

Despite the growing interest in AR optical combiners, most reported full‐color AR platforms are based on inorganic material systems [12, 13, 43] or liquid‐crystal‐based architectures [10, 11, 14] that generally rely on advanced fabrication processes. In this context, optical polymers for photonic platforms represent an attractive alternative: their transparency in the visible range, low cost, and compatibility with low‐temperature wet‐coating processes make them suitable for applications ranging from integrated optics to wearable photonics [44, 45]. Among the optical polymers, CYTOP [46], an amorphous fluorinated polymer with a low refractive index, has proven particularly useful as a cladding, substrate, or mold material, thanks to its chemical stability, optical transparency, and low surface energy, which also facilitates pattern demolding in imprint processes [46, 47, 48]. Nevertheless, demonstrations of fully polymeric GMR architectures simultaneously combining large‐area fabrication, environmental robustness, RGB‐selective operation, and high transparency remain scarce.

In this work, we present the design, fabrication, and experimental validation of a fully polymeric guided‐mode resonant metagrating specifically conceived for transparent AR applications. The proposed device is realized through a scalable thermal nanoimprint lithography process on a CYTOP–maN [49] platform and employs an encapsulated architecture in which the grating is embedded at the substrate–waveguide interface (see Figure 1a), thereby protecting the optical pattern from environmental exposure while preserving its resonant functionality. Unlike conventional diffraction gratings fabricated by imprinting techniques [36, 38] and polarization‐volume‐grating‐based AR combiners [10, 14], which inherently disperse different wavelengths into different directions, the proposed structure is engineered to generate three guided‐mode resonances corresponding to the primary RGB colors while reflecting them toward the same observation direction and maintaining high transparency over the remainder of the visible spectrum. The metagrating could be integrated into AR lenses to convey visual or textual cues toward the user's eye when illuminated by a projector [43, 50]. The main contributions of this work can be summarized as follows: (i) development and experimental validation of a complete fabrication strategy for large‐area fully polymeric guided‐mode resonant metagrating; (ii) implementation of an encapsulated architecture providing environmental protection and mechanical robustness while preserving the resonant optical response; (iii) engineering and experimental validation of simultaneous reflection of the three primary RGB colors toward the same observation direction under broadband illumination; and (iv) high transparency outside the resonant spectral bands. The combination of a fully polymeric platform, scalable fabrication, encapsulated architecture, RGB‐selective guided‐mode resonances, and same‐direction color routing provides a distinctive route toward cost‐effective optical combiners for augmented reality systems.

**FIGURE 1:**
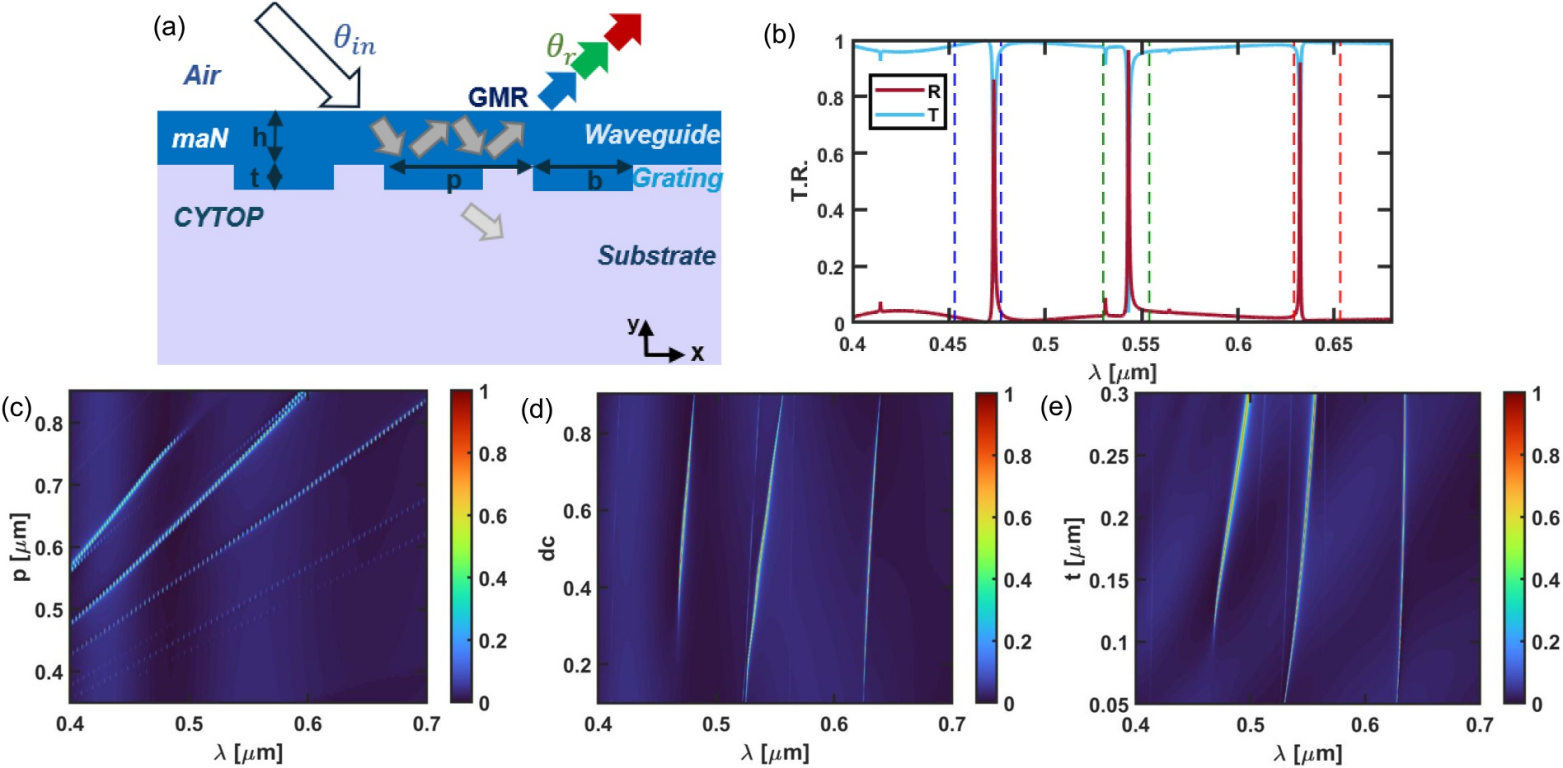
(a) Schematic cross‐section of the implemented guided‐mode resonant metagrating, consisting of a grating between a waveguide layer (both made of maN 2405) and a CYTOP substrate. Optimized geometric parameters: waveguide thickness h=0.495μm, grating thickness t=0.150μm, grating period p=0.740μm, grating ridge width b=0.444μm, duty cycle (defined as dc=b/p) dc=0.6, and incidence angle $\theta_{\text{in}}=45{}^{\circ}$ under TM polarization. (b) Simulated reflectance (red) and transmittance (light blue) spectra showing three guided‐mode resonance peaks located within narrow tolerance windows of ±12nm (vertical dashed lines) around the target wavelengths corresponding to the primary colors: blue (0.465μm), green (0.542μm), and red (0.641μm). The simulated resonance peaks are observed at 0.474μm, 0.545μm, and 0.633μm, respectively. (c–e) RCWA reflectance maps illustrating the evolution of the guided‐mode resonances as a function of wavelength and of (c) grating period p, (d) duty cycle dc, and (e) grating thickness t.

## 2 Design Based on Fabrication Constraints

The metagrating structure investigated in this work and shown in Figure 1a is based on a polymeric stack composed of multiple functional layers. From top to bottom, the configuration includes air as the superstrate and a waveguide on a grating, both made of maN 2405 [49] and placed on a CYTOP [46] substrate. The maN polymer is a negative and high‐refractive‐index resist (n>1.6 in the visible range) characterized by a strong absorption in the ultraviolet (UV) region; CYTOP has a low refractive index (n=1.339 at 580 nm), which provides an excellent low‐index cladding layer.

The materials and geometry were selected based on multiple criteria. First and foremost, it was necessary to identify a polymeric material pair exhibiting sufficient refractive‐index contrast to support three well‐separated guided‐mode resonances across the visible spectrum (400–700 nm) for an incident angle of 45°. The selected materials present refractive indices ranging from 1.33 to 1.68, allowing sufficient design flexibility for engineering the index contrast required for reliable GMR excitation [5, 7]. In addition, the use of polymeric materials was driven by the goal of leveraging thermal nanoimprint lithography as a cost‐effective and scalable fabrication technique. In NIL processes, CYTOP offers several advantages [32, 33]: its very low surface energy promotes clean, residue‐free demolding; its UV transparency makes it compatible with UV‐curable systems [30]; it is spin‐coatable and conformal, facilitating uniform deposition; and its chemical stability and hydrophobicity provide long‐term durability [34]. Importantly, its low glass transition temperature $\left(T_{g}\approx 108\;{}^{\circ}C\right)$ enables thermal imprinting at relatively low temperatures, avoiding polymer degradation [44, 45, 51]. A distinctive aspect of this work is the implemented configuration, here referred to as an encapsulated grating: the periodic profile is thermally imprinted into CYTOP, forming a negative grating pattern, which is subsequently backfilled with maN. This design provides three important advantages: (i) placing the grating between the substrate and the waveguide shields the pattern from contamination, degradation, and other environmental factors after fabrication; (ii) the use of CYTOP for thermal NIL enables reliable sub‐wavelength pattern transfer over large areas, leveraging its favorable surface properties; and (iii) it decouples the pattern definition from the UV response of maN, avoiding the strong UV absorption that would otherwise lead to nonuniform cross‐linking and hinder reproducible transfer of deep features. This approach therefore addresses environmental robustness and pattern‐transfer fidelity within a scalable fabrication flow.

Starting from these fabrication constraints and material considerations, the geometric parameters were optimized to support three guided‐mode resonances centered near the primary RGB wavelengths. The design objective was to obtain resonance peaks within a ±12 nm tolerance window around the nominal values for blue (465 nm), green (542 nm), and red (641 nm), with high transmittance in the rest of the visible spectrum. The spectral response was modeled using the rigorous coupled‐wave analysis (RCWA) method [52]. The simulation results (reflectance and transmittance spectra) shown in Figure 1b confirmed that a grating thickness t=150 nm, a waveguide thickness h=495 nm, a period p=740 nm, a grating ridge width b=444 nm, and a duty cycle (defined as dc=b/p) of 0.6 are optimal to simultaneously support three resonant modes in the visible when the structure is illuminated with an incident angle of $\theta_{\text{in}}=45{}^{\circ}$ under TM polarization. The simulated resonance peaks with the optimal geometry are observed at 0.474μm, 0.545μm, and 0.633μm. Furthermore, Figure 1b shows high transmittance across the rest of the visible spectrum, ensuring chromatic neutrality. Figure 1c–e reports RCWA parameter sweeps as a function of grating period, duty cycle, and grating depth. The grating period mainly controls the spectral position of the resonances, whereas duty cycle and depth primarily affect resonance tuning and coupling strength. The smooth evolution of the resonant features confirms the robustness of the selected design and indicates good tolerance to moderate fabrication‐induced dimensional variations. Table 1 summarizes, for each geometrical parameter, the optimal value and the corresponding tolerance range required to keep the three guided‐mode resonances within a ±20nm window around the target wavelengths.

**TABLE 1:** Sensitivity of the optimized geometrical parameters required to have the three guided‐mode resonances within a ±20nm wavelength window around the target RGB wavelengths.

Parameter	Optimal value	Tolerance range
Grating period, p	740nm	±20nm
Waveguide thickness, h	495nm	±30nm
Grating thickness, t	150nm	±20nm
Duty cycle, dc	0.6	±0.15

## 3 Fabrication Process

The overall fabrication workflow is illustrated in Figure 2, which outlines the main steps involved in the realization of the metagrating structures. The metagrating structures were fabricated on $2\times 2.5\;cm^{2}$ samples diced from 4‐inch Borofloat wafers, following a multistep protocol tailored for compatibility with nanoimprint lithography (NIL) on polymeric substrates.

**FIGURE 2:**
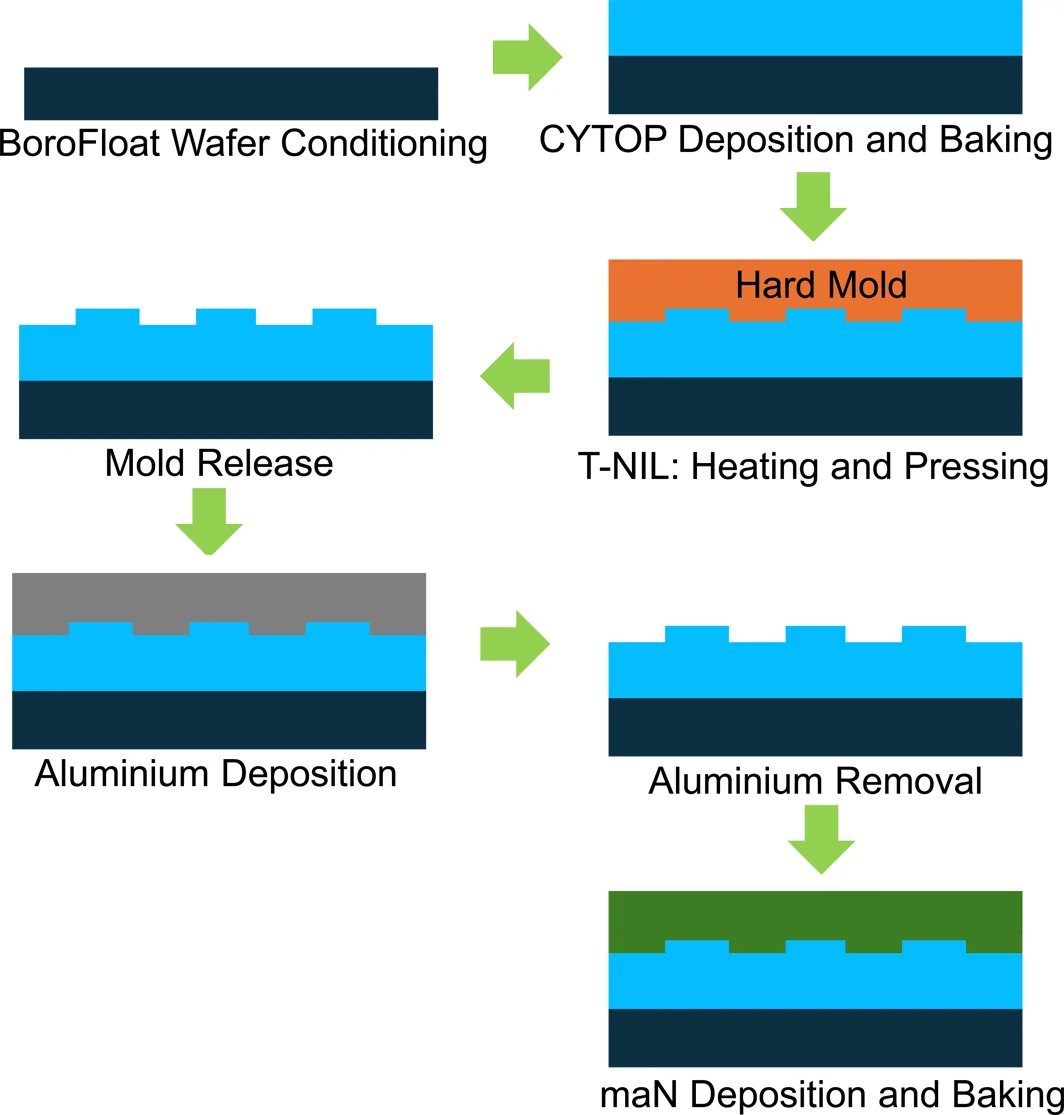
Overview of the multistep fabrication process of the metagrating structures. The process starts from Borofloat glass substrates, followed by CYTOP deposition and baking, nanoimprint lithography (NIL) using a hard mold to form the grating pattern on CYTOP. After demolding, an aluminum layer is thermally evaporated and subsequently removed by wet etching, resulting in a negative grating profile ready for functional layer deposition. Finally, maN is spin‐coated and baked.

Samples were cleaned (acetone, isopropanol [IPA], piranha for 3 min), activated by oxygen plasma treatment (Tepla 300) to promote adhesion. Before polymer deposition, each sample underwent a thermal conditioning step in an oven at 200°C for 10 min, to ensure complete dehydration and promote uniform polymer adhesion. Subsequently, CYTOP [46] (CTL‐809M, AGC Chemicals) was deposited by spin‐coating using a two‐step protocol: 500 rpm, 10 s, 200 rpm/s and then 2000 rpm, 30 s, 500 rpm/s. A final hotplate curing step was carried out to fully densify the film: 70°C for 30 min, 250°C for 120 min, and 70°C for 30 min. The resulting CYTOP layer was approximately 1μm thick (as measured using white light reflectance spectroscopy, FR‐ES by ThetaMetrisis), uniform, and transparent, thus forming a suitable imprintable base.

The shape of the negative grating was defined using T‐NIL performed on a Nanonex NX2000 tool. The NIL mold consisted of a silicon stamp with total dimensions of 2×2.5 ${\text{cm}}^{2}$ and a patterned grating area of 1.5×1.5 ${\text{cm}}^{2}$. The mold was fabricated by electron‐beam lithography followed by inductively coupled plasma reactive ion etching (ICP‐RIE) of silicon. Prior to lithography, a 200 nm‐thick layer of PMMA A5 resist was deposited on the silicon substrate by spin‐coating (4000 rpm, 2000 rpm/s, 60 s). The grating pattern was exposed using a LEICA EBPG 5000 electron‐beam lithography system operating at 100 keV, with an exposure dose of 780 μC/c$m^{2}$. The pattern was subsequently transferred into the silicon substrate by ICP‐RIE etching (Alcatel AMS4200). The etching was performed at 20°C–21°C for 1 min 11 s using a fluorine‐based plasma chemistry ($C_{4}F_{8}$/${SF}_{6}$/$O_{2}$), at a chamber pressure of $8\times 10^{-3}$ mbar, an ICP power of 450 W, and an RF bias power of 20 W. The process was optimized to target a grating depth of 150 nm. An anti‐sticking treatment was performed on the mold by depositing a monolayer of perfluorodecyltrichlorosilane (PDTS) for 5 min. To verify the effectiveness of the anti‐stick treatment, the static contact angle was measured using a Digidrop system, yielding a value of 107° and confirming a highly hydrophobic and nonwettable surface suitable for clean and reliable demolding. The fabricated silicon mold was characterized by both SEM (scanning electron microscopy) and AFM (atomic force microscopy) measurements. Figure 3a shows a representative SEM image acquired using a Verios 5 UC FE‐SEM. The image demonstrates the high uniformity of the fabricated mold structure: well‐defined grating ridges and the absence of visible large‐area defects within the inspected regions indicate a high‐fidelity replication of the intended geometry. Quantitative dimensional information used to evaluate the uniformity of the mold over the entire patterned area was subsequently obtained from AFM measurements (AFM Icon) performed at four different locations distributed across the grating surface, as reported in Figure 3b. The average geometrical dimensions extracted from the AFM measurements were a grating period of p=738.4±2.4 nm (mean ± standard deviation), a ridge width of b=443.3±5.0 nm, and a grating depth of t=149.7±1.4 nm, showing excellent agreement with the target design values of 740, 444, and 150 nm, respectively. The low standard deviations observed for all geometrical parameters confirm the excellent uniformity of the fabricated mold over the entire patterned area and indicate a highly controlled fabrication process.

**FIGURE 3:**
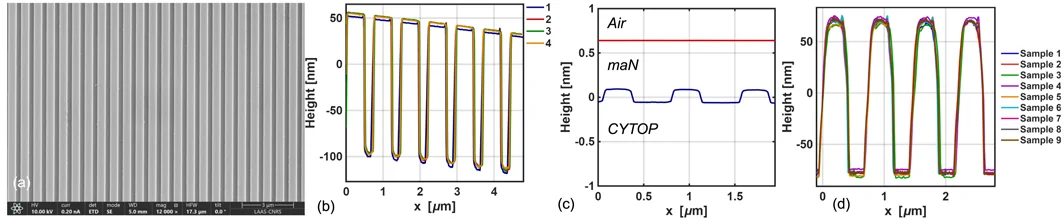
(a) Top‐view SEM (scanning electron microscopy) image of the fabricated silicon mold acquired using a Verios 5 UC FE‐SEM. (b) Atomic force microscopy (AFM) line scans of the mold performed acquired using an AFM Icon system at four different locations across the patterned area. (c) Atomic force microscopy line scans of the structure. The blue trace corresponds to the grating profile obtained after thermal nanoimprint lithography, using optimized parameters: 180°C, 240 PSI, 2 min. The red trace represents the AFM scan repeated after maN deposition, confirming a flat and uniform top surface. (d) AFM profiles measured at the center of nine independently fabricated samples. Average geometrical parameters of p=741.0±5.3 nm, b=441.7±5.8 nm, and t=148.2±2.9 nm (mean ± standard deviation).

Multiple imprint recipes were evaluated and empirically optimized to ensure stable operation and reproducible pattern transfer. The final optimized imprint parameters, 180°C, 240 PSI (pounds per square inch), for 2 minutes, were identified through iterative testing as the best compromise between sufficient thermal activation of CYTOP and the mechanical fidelity required for accurate pattern replication. Lower imprint temperatures resulted in incomplete pattern transfer due to insufficient polymer flow, whereas excessively high temperatures led to partial melting of the structure, compromising the integrity of the imprinted grating. In terms of pressure, higher values exceeded the stable operational range of the tool and increased the risk of mechanical damage to both the sample and the mold, particularly during demolding. Conversely, low pressures were insufficient to ensure full feature replication, often resulting in incomplete or nonuniform imprint profiles. The imprinted features were characterized by atomic force microscopy (AFM Icon). AFM measurements were performed at four different locations distributed across the patterned area to evaluate the uniformity of the replicated grating. The extracted geometrical parameters yielded average values of grating period p=743.7±6.1 nm, ridge width b=446.3±5.7 nm, and grating depth t=148.2±1.5 nm (mean ± standard deviation), demonstrating excellent uniformity of the nanoimprinted structures over the entire device area. The reproducibility of the fabrication process was further assessed by performing AFM measurements on nine independently fabricated samples. Figure 3d shows the overlap of the corresponding AFM profiles acquired at the center of each sample. Despite being fabricated in independent imprint runs, the measured profiles exhibit excellent agreement. The average geometrical parameters extracted from the nine samples were *p* = 741.0 ± 5.3 nm, *b* = 441.7 ± 5.8 nm, and *t* = 148.2 ± 2.9 nm (mean ± standard deviation), confirming the high reproducibility of the fabrication process.

Following the nanoimprint process, a 200 nm‐thick aluminum (Al) layer was thermally evaporated onto the patterned CYTOP surface and subsequently removed by wet etching. This step proved essential to overcome a critical limitation of CYTOP in subsequent processing: its intrinsically low surface energy renders it highly resist‐phobic, hindering the uniform deposition and adhesion of polymeric resists such as maN 2405. Without the introduction of a metallic interlayer, spin‐coated resists were prone to dewetting and droplet formation. To overcome this problem, it has been shown [47, 48] that by first sputtering Al on the CYTOP surface, and subsequently removing it by chemical etching, the surface properties are modified and enable proper adhesion of organic resists. A thickness of 200 nm was found to provide the best compromise between conformal coverage and clean etching. The aluminum was deposited by thermal evaporation (Eva 600) using a controlled power ramp to prevent film stress and to ensure adhesion uniformity across the sample. After deposition, the Al layer was removed using a heated wet chemical solution ($H_{3}{\text{PO}}_{4}$, ${\text{HNO}}_{3}$, $H_{2}$O) at 50°C for 1 minute. The short etch duration is sufficient to ensure complete removal of the metallic layer without degradation of the underlying polymer. To assess whether the grating profile was altered by the metal coating or the etching step, AFM scans were repeated immediately after Al removal, showing that the structural parameters were fully preserved, matching the pre‐deposition measurements. This confirms the mechanical robustness of the CYTOP grating and the noninvasive nature of this Al process.

The high‐index grating and waveguide layers were respectively backfilled and formed via spin‐coating of maN [49] (micro resist technology). Due to the nonflat (patterned) surface, several tests were carried out to calibrate the spin speed and ensure uniform coating over both trenches and ridges. As a first step, maN thickness was measured on flat substrates at different spin speeds to establish baseline behavior. Subsequently, spin‐coating tests were performed on patterned surfaces with 150 nm‐deep trenches to determine the correction factor needed to compensate for the nonplanarity. The resist thickness on corrugated samples was obtained by the difference from the thickness data of the different layers. The optimal condition was identified as 2600 rpm for 60 s with an acceleration of 4000 rpm/s, resulting in a final thickness of 490nm±10nm that was uniform across the structured area. After spin‐coating, the sample was baked on a hotplate at 90°C for 1 min to remove residual solvents without affecting the trench filling. An AFM scan was then repeated to assess the surface morphology. The red trace in Figure 3c shows the profile after maN deposition, confirming that the material produced a flat and homogeneous top surface.

To stabilize the maN and increase chemical resistance, some of the coated samples were exposed to UV light using a Kube 9 lamp for varying durations (5–10–15 min). It was observed that maN exhibits strong absorption in the UV, leading to a visible yellowish tint and slightly reduced transparency of the final device. Spectrophotometric analysis reveals that maN absorbance gradually increases with UV exposure time, particularly in the 200–400 nm wavelength range. This increase is associated with photoinduced cross‐linking in the resist. Beyond 400 nm, the absorbance tends to converge toward zero regardless of exposure time, indicating minimal optical impact in the visible region. Based on these observations, an exposure time of 10 min was selected as a compromise: it is long enough to promote adequate cross‐linking and mechanical stability for subsequent processing while keeping the optical absorption within acceptable limits for transparent device operation.

## 4 Results and Discussion

To evaluate the optical response of the fabricated samples, spectroscopic ellipsometry measurements were performed using an EP4 ellipsometer in reflection configuration (Figure 4e), where the incident polarized beam impinges on the sample at an angle of $\theta_{\text{in}}=45{}^{\circ}$, and the reflected light is collected by the detection module at $\theta_{r}=-45{}^{\circ}$. This technique provides the ellipsometric angles Ψ and Δ, which describe the amplitude ratio and phase difference between the p‐ and s‐polarized components of the reflected light, respectively. The spectral measurements were acquired across the visible range at an incidence angle of 45°. In these measurements, the grating K‐vector was manually aligned along the p‐plane of incidence.

**FIGURE 4:**
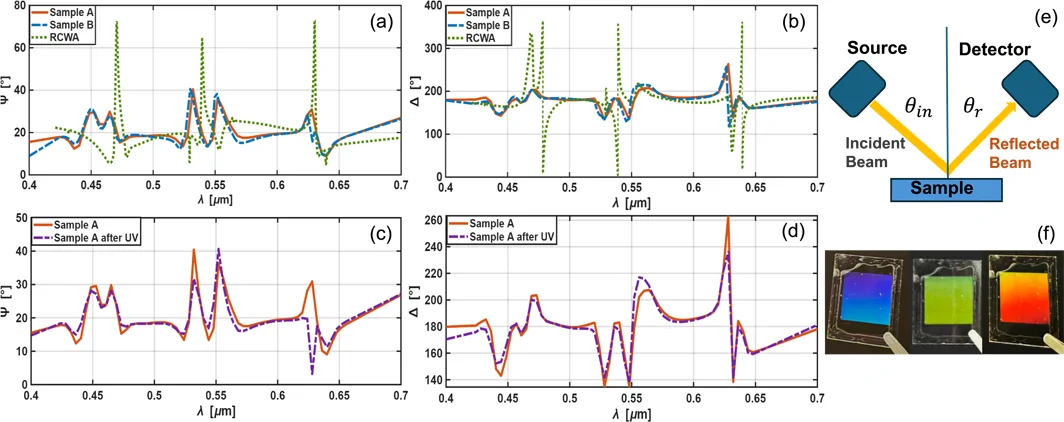
Ellipsometric curves for Ψ (a) and Δ (b) measured for two different samples, Sample A (orange solid line) and Sample B (blue dash‐dotted line), fabricated following the same protocol. Measurements are compared with RCWA simulations (green dotted line). Panels (c) and (d) show Ψ and Δ, respectively, for Sample A before (orange solid line) and after (violet dash‐dotted line) the 10‐min UV exposure. (e) Schematic representation of the measurement geometry used in the EP4 ellipsometer setup. (f) Photographs of the sample when illuminated with polarized white light at a decreasing angle (from left to right), showing the reflection of blue, green, and red.

Two independently fabricated samples (Sample A and Sample B) were characterized, both before and after UV exposure of the maN layer, to assess fabrication reproducibility and compare measurements with RCWA simulations. The experimental results shown in Figure 4a–d reveal three distinct features in both Ψ and Δ curves, which correspond to the expected guided‐mode resonances predicted by simulations. The resonance wavelengths are centered around 459 nm, 545 nm, and 628 nm, consistent with the design targets for blue, green, and red selective reflection. The agreement between the measured and simulated (green dotted line) resonance positions, shown in Figure 4a,b, confirms the successful optical operation of the fabricated metagratings. Furthermore, despite the different fabrication runs, Sample A (solid orange line) and Sample B (blue dash‐dotted line), which were fabricated using the same protocol, exhibit overlapping Ψ and Δ curves. This indicates a high degree of reproducibility and reliability in the fabrication process, confirming that the same design and fabrication protocol developed in this work yields consistent results across different samples. Figure 4c,d show that Sample A maintains nearly the same spectral behavior both before (orange solid line) and after (violet dash‐dotted line) UV exposure for 10 minutes. This suggests that the UV treatment does not significantly alter the optical properties of Sample A, ensuring its stability across different processing conditions. To further assess device stability, additional ellipsometric measurements were performed approximately 1 year after the original characterization. The spectra exhibited excellent agreement with the initial measurements, with no significant shifts in the resonance positions or degradation of the optical response, confirming the long‐term stability of the encapsulated polymeric metagratings under ambient conditions.

The results also highlight the effectiveness of NIL in achieving transparent, polymeric metagratings with nanometric precision. This is particularly relevant given the challenges of patterning polymer materials with high fidelity over large areas. In some measurements, a weak splitting of the resonance peaks was observed. This phenomenon may result from imperfect polarization states due to a slight misalignment between the grating and measurement axes, leading to a mixed TE–TM excitation. This is particularly relevant since the Ψ angle represents the amplitude ratio between TM and TE components. Further investigation is ongoing to explore these effects through extended simulation and angle‐resolved measurements. Overall, the fabricated devices exhibit the intended trichromatic response with good agreement with the design targets, whereas the reflected light shows highly saturated and well‐defined RGB colors (Figure 4f), demonstrating the effectiveness of the proposed fabrication strategy for AR applications. To further validate the simulated optical response, preliminary angle‐dependent reflectance measurements were performed under TM polarization at incidence angles of 40°, 45°, and 50°. To experimentally determine the resonance wavelengths, the sample was illuminated with a broadband light source, whereas the reflected light was collected using a silicon photodetector mounted on a rotating detection arm. The measured resonance wavelengths were then compared with the corresponding RCWA predictions. Figure 5 shows the comparison between the experimentally determined resonance wavelengths and the RCWA‐simulated reflectance map as a function of wavelength and the incidence angle. The measured resonance positions closely follow the simulated dispersion branches, confirming the predicted angular evolution of the guided‐mode resonances. The angular wavelength shift rates obtained from the simulations were 5.4nm/°, 6.6nm/°, and 7.5nm/° for the three resonance branches, whereas the experimentally measured values for the two resonance branches observed over the investigated angular range were 5.9nm/° and 7.7nm/°, respectively, confirming good agreement with the simulated angular behavior. A comprehensive angle‐resolved optical characterization will be addressed in future work.

**FIGURE 5:**
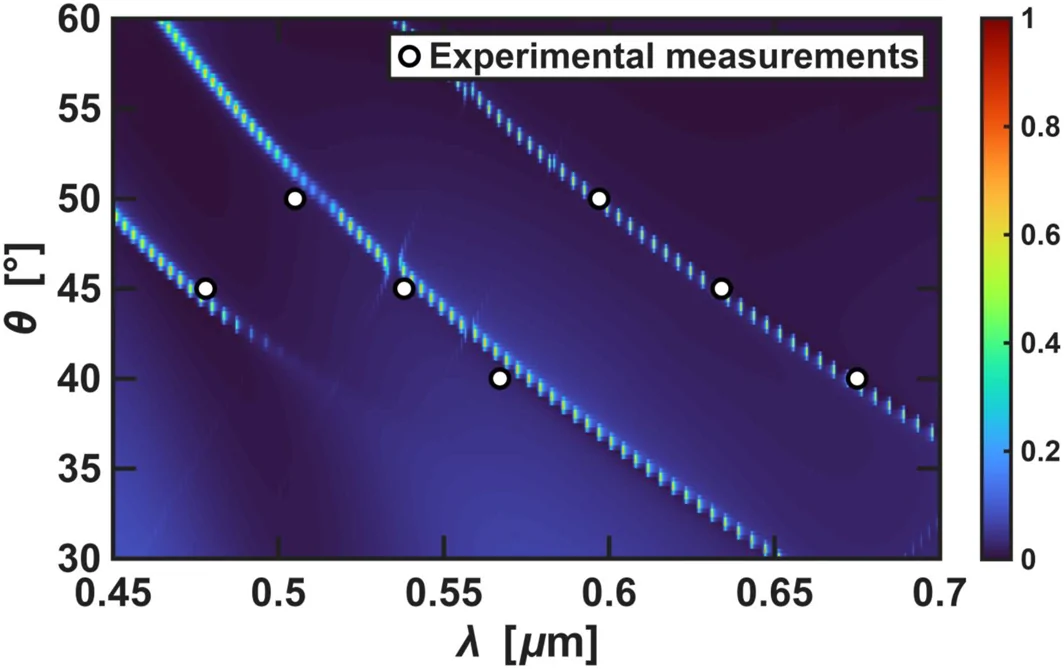
RCWA‐simulated reflectance map as a function of wavelength and the incidence angle under TM polarization. Circular markers indicate the resonance wavelengths experimentally determined from angle‐dependent reflectance measurements performed at incidence angles of 40°, 45°, and 50°.

## 5 Conclusions

In this work, we demonstrated a scalable fabrication strategy for fully polymeric guided‐mode resonant metagratings designed for wavelength‐selective reflection in the visible range and targeting augmented reality applications. Starting from an RCWA‐optimized design, we realized a transparent encapsulated grating architecture in which the resonant structure is embedded at the substrate–waveguide interface, providing intrinsic environmental protection while preserving the desired optical response. The proposed fabrication process was systematically developed and experimentally validated through each processing step, from mold fabrication and thermal nanoimprint lithography to polymer backfilling and waveguide fabrication. SEM and AFM characterizations confirmed the successful transfer of the designed geometry, high structural fidelity, and excellent uniformity over the patterned area. Furthermore, quantitative AFM measurements performed on multiple independently fabricated samples demonstrated the high reproducibility and robustness of the proposed fabrication approach. Optical characterization by spectroscopic ellipsometry confirmed the presence of three guided‐mode resonances close to the target RGB wavelengths, showing good agreement with RCWA simulations. Preliminary angle‐dependent reflectance measurements further validated the simulated angular evolution of the resonances, whereas long‐term ellipsometric measurements demonstrated the stability of the encapsulated polymeric architecture under ambient conditions. Future work will focus on performing extended optical characterization to fully map the optical response of the fabricated devices. The results demonstrate that thermal nanoimprint lithography provides a robust, scalable, and cost‐effective fabrication route for transparent polymeric guided‐mode resonant metagratings while preserving excellent structural fidelity and reliable optical performance. The proposed fabrication strategy therefore represents a promising platform for the realization of large‐area GMR‐based photonic devices for augmented reality and other wavelength‐selective photonic applications.

## Author Contributions

**Francesca Filograno:** conceptualization, investigation, writing – original draft, methodology, validation, writing – review and editing, software, data curation, project administration, resources, visualization. **Olivier Gauthier‐Lafaye:** conceptualization, investigation, writing – review and editing, methodology, validation, supervision, data curation, project administration, resources, software, visualization. **Adrian Laborde:** methodology, investigation, resources. **Jean‐Baptiste Doucet:** methodology, investigation, resources. **Vincent Raimbault:** investigation, methodology, resources. **Nathalie Bardou:** methodology, investigation, resources. **Béatrice Dagens:** investigation, methodology, writing – review and editing, supervision, validation, resources, visualization. **Giovanna Calò:** conceptualization, investigation, writing – review and editing, methodology, validation, software, supervision, data curation, project administration, resources, visualization. **Giovanni Magno:** conceptualization, investigation, writing – review and editing, methodology, validation, software, supervision, data curation, project administration, resources, visualization.

## Conflicts of Interest

The authors declare no conflicts of interest.

## Data Availability

The datasets generated during and/or analyzed during the current study are available from the corresponding author on reasonable request.
